# Response to “Enhancing the XGBoost Mortality Prediction Model for ICU Patients with Acute Ischemic Stroke”

**DOI:** 10.34133/hds.0394

**Published:** 2025-12-09

**Authors:** Jack A. Cummins, Ben S. Gerber, Mayuko Ito Fukunaga, Nils Henninger, Catarina I. Kiefe, Feifan Liu

**Affiliations:** ^1^ Manchester Essex Regional High School, Manchester, MA 01944, USA.; ^2^ Department of Population and Quantitative Health Sciences, UMass Chan, Worcester, MA 01665, USA.; ^3^ Division of Health Informatics and Implementation Science, UMass Chan, Worcester, MA 01655, USA.; ^4^ Division of Pulmonary, Allergy and Critical Care Medicine, UMass Chan, Worcester, MA 01655, USA.; ^5^ Meyers Primary Care Institute, Worcester, MA 01605, USA.; ^6^ Department of Neurology, UMass Chan, Worcester, MA 01655, USA.

We sincerely appreciate the insightful comments and suggestions by Meng and colleagues [1] for our recently published paper, entitled “In-hospital mortality prediction among intensive care unit patients with acute ischemic stroke: A machine learning approach” [2]. We are pleased that our emphasis on model fairness, interpretability, and comprehensive benchmarking were found valuable, and their perspectives are very inspirational on expanding and enhancing our work.

We agree that external validation is a critical next step toward ensuring model reproducibility and clinical generalizability. As noted in our Discussion section, we plan to validate the model using the eICU Collaborative Research Database, which contains multi-institutional data from over 200 hospitals across the United States. The authors correctly point out in the letter that the clarification of preprocessing details [specifically, the inclusion criteria for the electronic intensive care unit (eICU) dataset and any additional preprocessing that may be necessary] is essential for model reproducibility and future external validation. Our future validation protocol will therefore explicitly document cohort selection [e.g., International Classification of Diseases-10 (ICD-10)/ICD-9 criteria], missing-data imputation, and temporal feature aggregation to enable transparent replication and benchmarking by other investigators.

We appreciate the authors’ recognition of the prognostic importance of Glasgow coma scale (GCS) and blood urea nitrogen (BUN) levels. Their cited work [3] further reinforces our observation that the interactive nature of neurologic and metabolic factors captures acute physiologic deterioration in ischemic stroke patients. We also concur that exploring feature interactions, such as the effect of high BUN levels and low GCS levels, could yield more granular risk stratification and align with current interpretability frameworks such as Shapley Additive Explanation (SHAP) interaction values.

The integration of real-time electronic health record (EHR) data is a highly attractive suggestion that the authors propose. Transitioning from static models trained on 48-h summaries to a dynamic, continuously updated model would be very useful to support time-sensitive decision support. We envision combining tree-based models with sequence architectures (e.g., gated recurrent units or Transformer-based encoders) to model temporal dependencies while maintaining interpretability. Such integration may facilitate adaptive clinical alerts and more responsive resource allocation in the ICU setting.

We also agree that the prediction horizon of the model could be expanded to include the outcomes after discharge (e.g., 30- and/or 90-d mortality, readmission, and functional recovery), because it would improve the model’s clinical utility and the care continuity. While the current Medical Information Mart for Intensive Care-IV (MIMIC-IV) dataset lacks direct post-discharge linkages, we are exploring methods to couple it with follow-up or claims-based data to analyze the longitudinal course of stroke patients.

We are very grateful for all the insightful comments by Meng and his team, which provides valuable guidance for extending our model’s reproducibility, interpretability, and clinical utility. It also aligns well with our overarching goal of developing ethically responsible, interpretable, and generalizable machine-learning models to improve patient care. We look forward to future cross-institutional collaborations that will further advance data-driven precision medicine for patients admitted with acute ischemic stroke.
